# 2-{[4-(Phenyldiazenyl)phenyl]iminomethyl}phenol

**DOI:** 10.1107/S1600536808027244

**Published:** 2008-08-30

**Authors:** Hoong-Kun Fun, Reza Kia, Stefanie Schiffers, Majid Moghadam, Paul R. Raithby

**Affiliations:** a X-ray Crystallography Unit, School of Physics, Universiti Sains Malaysia, 11800 USM, Penang, Malaysia; b Chemistry Department, University of Bath, Claverton Down, Bath BA2 7AY, UK; c Chemistry Department, University of Isfahan, Isfahan 81746-73441, Iran

## Abstract

The mol­ecule of the title compound, C_19_H_15_N_3_O, is approximately planar and displays a *trans* configuration with respect to the C=N and N=N double bonds. An intra­molecular O—H⋯N hydrogen bond generates an *S*(6) ring motif. The dihedral angles between the hydroxy­phenyl ring and the phenyl and benzene rings are 4.31 (8) and 6.60 (8)°, respectively. The dihedral angle between the phenyl and benzene rings linked by the azo group is 2.70 (8)°. The imino group is coplanar with the hydroxy­phenyl ring, as shown by the C—C—C—N torsion angle of −1.8 (2)°. The azo group is disordered over two position with refined site-occupancy factors of *ca* 0.87/0.13. In the crystal structure, mol­ecules are linked together by inter­molecular C—H⋯O inter­actions along the *c* axis and also are packed as one-dimensional extended chains down the *b* axis.

## Related literature

For bond-length data, see: Allen *et al.* (1987 ▶). For hydrogen-bond motifs, see: Bernstein *et al.* (1995 ▶). For related structures, see: Vani & Vijayan (1977 ▶); Revannasiddaiah *et al.* (1997 ▶). For background to the applications, see, for example: Liu *et al.* (1990 ▶); Ikeda & Tsutsumi (1995 ▶); Evans *et al.* (1980 ▶); Griffiths & Allen *et al.* (1980 ▶); Flamingi & Monti (1985 ▶); Leaver *et al.* (1980 ▶).
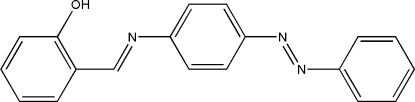


## Experimental

### 

#### Crystal data

C_19_H_15_N_3_O
*M* *_r_* = 301.34
Monoclinic, 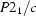

*a* = 26.0537 (10) Å
*b* = 4.5475 (2) Å
*c* = 12.0423 (4) Å
β = 90.600 (2)°
*V* = 1426.68 (10) Å^3^
*Z* = 4
Mo *K*α radiation
μ = 0.09 mm^−1^
*T* = 100.0 (1) K
0.52 × 0.20 × 0.06 mm

#### Data collection

Bruker SMART APEXII CCD area-detector diffractometer
Absorption correction: multi-scan (*SADABS*; Bruker, 2005 ▶) *T*_min_ = 0.955, *T*_max_ = 0.995
35506 measured reflections
4662 independent reflections
3356 reflections with *I* > 2σ(*I*)
*R*_int_ = 0.053

#### Refinement

*R*[*F* ^2^ > 2σ(*F* ^2^)] = 0.071
*wR*(*F* ^2^) = 0.212
*S* = 1.08
4662 reflections
220 parameters
H atoms treated by a mixture of independent and constrained refinement
Δρ_max_ = 0.88 e Å^−3^
Δρ_min_ = −0.53 e Å^−3^

### 

Data collection: *APEX2* (Bruker, 2005 ▶); cell refinement: *APEX2*; data reduction: *SAINT* (Bruker, 2005 ▶); program(s) used to solve structure: *SHELXTL* (Sheldrick, 2008 ▶); program(s) used to refine structure: *SHELXTL*; molecular graphics: *SHELXTL*; software used to prepare material for publication: *SHELXTL* and *PLATON* (Spek, 2003 ▶).

## Supplementary Material

Crystal structure: contains datablocks global, I. DOI: 10.1107/S1600536808027244/at2620sup1.cif

Structure factors: contains datablocks I. DOI: 10.1107/S1600536808027244/at2620Isup2.hkl

Additional supplementary materials:  crystallographic information; 3D view; checkCIF report

## Figures and Tables

**Table 1:** Hydrogen-bond geometry (Å, °)

*D*—H⋯*A*	*D*—H	H⋯*A*	*D*⋯*A*	*D*—H⋯*A*
O1—H1*O*1⋯N1	1.01 (3)	1.66 (2)	2.5853 (18)	150 (2)
C5—H5*A*⋯O1^i^	0.93	2.59	3.386 (2)	144

Symmetry code: (i) 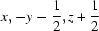.

**Fig. 1.**
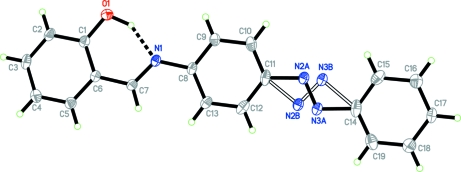
The molecular structure of (I), showing 50% probability displacement ellipsoids. Intramolecular hydrogen bond is shown as a dashed line. Open bonds indicate the minor disordered component.

**Fig. 2.**
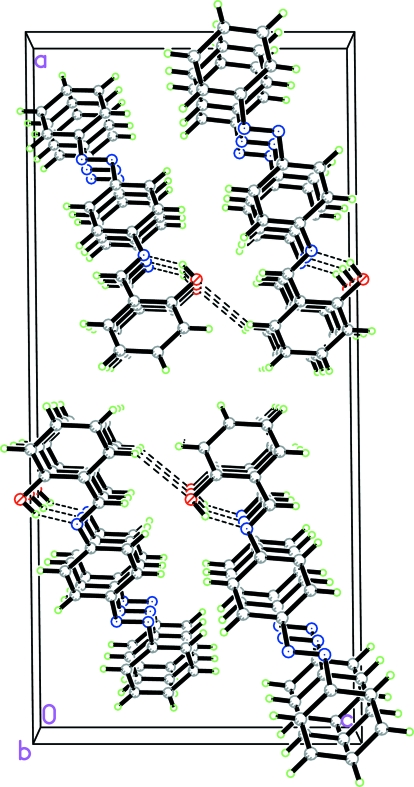
The crystal packing of the major component of (I), viewed down the b-axis, showing stacking of molecules. Intramolecular and intermolecular interactions are shown as dashed lines.

**Fig. 3.**
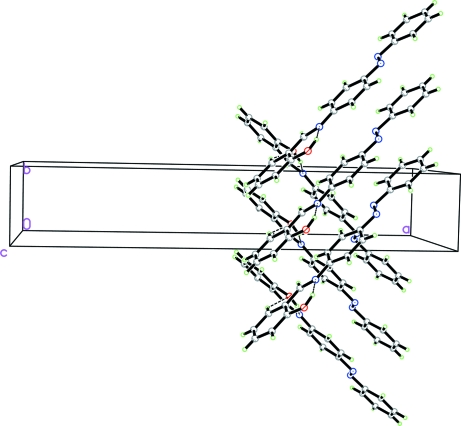
The crystal structure of the major component of (I), showing 1-D extended chains along the b-axis. Intramolecular and intermolecular interactions are shown as dashed lines.

## supplementary crystallographic information

### Comment

Azobenzene and its derivatives have attracted much attention for their high potential in industrial applications, such as liquid crystals, light-driven switches and image-storage devices (Liu *et al.*, 1990; Ikeda & Tsutsumi, 1995). In addition, azo compounds represent the dominant class of synthetic colourant employed in the textile, printing, agrochemical and pharmaceutical industries. As a result of the presence of the stable chromophoric azo group (N═N) which is capabale of linking different aromatic systems with electron-donating and/or electron-withdrawing groups, dyes can be designed to resist chemical or photochemical degradation processes (Evans *et al.*, 1980; Griffiths & Allen, 1980; Leaver *et al.*, 1980; Flamingi & Monti, 1985).

In the title compound (I) (Fig. 1), the molecule adopts a *trans* configuration with respect to the C═N and N═N double bonds. The bond lengths and angles are within the normal ranges (Allen *et al.*, 1987). An intramolecular O—H···N hydrogen bond generates a *S*(6) ring motif (Bernstein *et al.*, 1995). The dihedral angles between the hydroxyphenyl ring and the two phenyl rings are 4.31 (8) and 6.60 (8)°, respectively. The dihedral angle between the two phenyl rings joined by the azo group is 2.70 (8)°. The azo group is disordered over two position and the refined site-occupancy factors of the disordered parts are 0.869 (3)/0.131 (3). The imino group is coplanar with the hydroxyohenyl ring as it can be shown by the C1—C6—C7—N1 torsion angle of -1.8 (2)°. In the crystal structure, molecules are linked together by intermolecular C—H···O interactions (Table 1) along the *c* axis and also are packed as 1-D extended chains down the *b* axis (Fig. 2).

### Experimental

The title compound was synthesized by mixing equimolar amount of the *p*-phenylazo aniline and salicylaldehyde in ethanol under reflux condition for 1 h. Single crystals suitable for *X*-ray diffraction were obtained by evaporation of an ethanol solution at room temperature.

### Refinement

The H atom bound to the oxygen atom was located from the difference Fourier map and refined freely with the parent atom. The rest of the hydrogen atoms were positioned geometrically and refined as riding model with C—H = 0.93 Å and *U*_iso_(H) = 1.2*U*_eq_(C). The highest peak is located 0.73 Å from C11 and the deepest hole is located 0.66 Å from C19.

### Crystal data

**Table:**

C_19_H_15_N_3_O	*F*_000_ = 632
*M**_r_* = 301.34	*D*_x_ = 1.403 Mg m^−^^3^
Monoclinic, *P*2_1_/*c*	Mo *K*α radiation λ = 0.71073 Å
Hall symbol: -P 2ybc	Cell parameters from 4872 reflections
*a* = 26.0537 (10) Å	θ = 3.1–31.1º
*b* = 4.5475 (2) Å	µ = 0.09 mm^−^^1^
*c* = 12.0423 (4) Å	*T* = 100.0 (1) K
β = 90.600 (2)º	Plate, yellow
*V* = 1426.68 (10) Å^3^	0.52 × 0.20 × 0.06 mm
*Z* = 4	

### Data collection

**Table:**

Bruker SMART APEXII CCD area-detector diffractometer	4662 independent reflections
Radiation source: fine-focus sealed tube	3356 reflections with *I* > 2σ(*I*)
Monochromator: graphite	*R*_int_ = 0.053
*T* = 100.0(1) K	θ_max_ = 31.4º
φ and ω scans	θ_min_ = 0.8º
Absorption correction: multi-scan(SADABS; Bruker, 2005)	*h* = −38→38
*T*_min_ = 0.955, *T*_max_ = 0.995	*k* = −6→6
35506 measured reflections	*l* = −17→17

### Refinement

**Table:**

Refinement on *F*^2^	Secondary atom site location: difference Fourier map
Least-squares matrix: full	Hydrogen site location: inferred from neighbouring sites
*R*[*F*^2^ > 2σ(*F*^2^)] = 0.071	H atoms treated by a mixture of independent and constrained refinement
*wR*(*F*^2^) = 0.212	*w* = 1/[σ^2^(*F*_o_^2^) + (0.1093*P*)^2^ + 0.6819*P*] where *P* = (*F*_o_^2^ + 2*F*_c_^2^)/3
*S* = 1.08	(Δ/σ)_max_ < 0.001
4662 reflections	Δρ_max_ = 0.88 e Å^−^^3^
220 parameters	Δρ_min_ = −0.52 e Å^−^^3^
Primary atom site location: structure-invariant direct methods	Extinction correction: none

### Special details

**Table:**

Experimental. The low-temperature data was collected with the Oxford Cyrosystem Cobra low-temperature attachment.
Geometry. All e.s.d.'s (except the e.s.d. in the dihedral angle between two l.s. planes) are estimated using the full covariance matrix. The cell e.s.d.'s are taken into account individually in the estimation of e.s.d.'s in distances, angles and torsion angles; correlations between e.s.d.'s in cell parameters are only used when they are defined by crystal symmetry. An approximate (isotropic) treatment of cell e.s.d.'s is used for estimating e.s.d.'s involving l.s. planes.
Refinement. Refinement of *F*^2^ against ALL reflections. The weighted *R*-factor *wR* and goodness of fit *S* are based on *F*^2^, conventional *R*-factors *R* are based on *F*, with *F* set to zero for negative *F*^2^. The threshold expression of *F*^2^ > σ(*F*^2^) is used only for calculating *R*-factors(gt) *etc*. and is not relevant to the choice of reflections for refinement. *R*-factors based on *F*^2^ are statistically about twice as large as those based on *F*, and *R*- factors based on ALL data will be even larger.

### Fractional atomic coordinates and isotropic or equivalent isotropic displacement parameters (Å^2^)

**Table:**

	*x*	*y*	*z*	*U*_iso_*/*U*_eq_	Occ. (<1)
O1	0.34931 (5)	−0.2401 (3)	0.48842 (10)	0.0256 (3)	
N1	0.31269 (5)	0.0728 (3)	0.64837 (11)	0.0163 (3)	
N2A	0.16497 (6)	0.9300 (4)	0.75288 (13)	0.0178 (4)	0.869 (3)
N3A	0.16275 (6)	1.0337 (4)	0.85014 (13)	0.0181 (4)	0.869 (3)
N2B	0.1809 (4)	0.924 (3)	0.8304 (9)	0.0178 (4)	0.131 (3)
N3B	0.1469 (5)	1.036 (3)	0.7639 (9)	0.0181 (4)	0.131 (3)
C1	0.38180 (6)	−0.3582 (4)	0.56362 (13)	0.0188 (3)	
C2	0.41797 (7)	−0.5647 (4)	0.52832 (14)	0.0226 (4)	
H2A	0.4196	−0.6172	0.4538	0.027*	
C3	0.45127 (7)	−0.6905 (4)	0.60503 (15)	0.0240 (4)	
H3A	0.4757	−0.8247	0.5811	0.029*	
C4	0.44882 (6)	−0.6195 (4)	0.71718 (14)	0.0225 (4)	
H4A	0.4709	−0.7086	0.7681	0.027*	
C5	0.41316 (6)	−0.4151 (4)	0.75211 (14)	0.0202 (3)	
H5A	0.4115	−0.3668	0.8270	0.024*	
C6	0.37947 (6)	−0.2794 (3)	0.67662 (13)	0.0164 (3)	
C7	0.34330 (6)	−0.0621 (4)	0.71608 (13)	0.0169 (3)	
H7A	0.3421	−0.0191	0.7915	0.020*	
C8	0.27744 (6)	0.2872 (3)	0.68533 (13)	0.0165 (3)	
C9	0.24374 (6)	0.3987 (4)	0.60503 (14)	0.0197 (3)	
H9A	0.2457	0.3306	0.5324	0.024*	
C10	0.20753 (6)	0.6087 (4)	0.63155 (15)	0.0227 (4)	
H10A	0.1852	0.6789	0.5770	0.027*	
C11	0.20438 (6)	0.7148 (4)	0.73884 (15)	0.0224 (4)	
C12	0.23831 (7)	0.6073 (4)	0.82083 (15)	0.0236 (4)	
H12A	0.2365	0.6783	0.8931	0.028*	
C13	0.27454 (6)	0.3952 (4)	0.79410 (13)	0.0199 (3)	
H13A	0.2969	0.3248	0.8485	0.024*	
C14	0.12253 (7)	1.2474 (4)	0.86184 (16)	0.0252 (4)	
C15	0.08839 (7)	1.3377 (4)	0.77750 (16)	0.0270 (4)	
H15A	0.0908	1.2591	0.7065	0.032*	
C16	0.05072 (7)	1.5467 (4)	0.80128 (15)	0.0256 (4)	
H16A	0.0279	1.6082	0.7460	0.031*	
C17	0.04748 (6)	1.6621 (4)	0.90783 (15)	0.0230 (4)	
H17A	0.0222	1.7995	0.9242	0.028*	
C18	0.08194 (7)	1.5724 (4)	0.98979 (15)	0.0254 (4)	
H18A	0.0798	1.6515	1.0608	0.031*	
C19	0.11925 (7)	1.3670 (4)	0.96659 (16)	0.0263 (4)	
H19A	0.1423	1.3090	1.0219	0.032*	
H1O1	0.3277 (10)	−0.093 (6)	0.530 (2)	0.051 (7)*	

### Atomic displacement parameters (Å^2^)

**Table:**

	*U*^11^	*U*^22^	*U*^33^	*U*^12^	*U*^13^	*U*^23^
O1	0.0321 (7)	0.0284 (7)	0.0164 (5)	0.0085 (5)	0.0000 (5)	−0.0010 (5)
N1	0.0171 (6)	0.0130 (6)	0.0188 (6)	−0.0002 (5)	0.0030 (5)	−0.0009 (5)
N2A	0.0183 (8)	0.0145 (8)	0.0206 (7)	0.0000 (6)	0.0030 (6)	−0.0009 (6)
N3A	0.0181 (8)	0.0152 (8)	0.0212 (7)	0.0002 (6)	0.0036 (6)	−0.0018 (6)
N2B	0.0183 (8)	0.0145 (8)	0.0206 (7)	0.0000 (6)	0.0030 (6)	−0.0009 (6)
N3B	0.0181 (8)	0.0152 (8)	0.0212 (7)	0.0002 (6)	0.0036 (6)	−0.0018 (6)
C1	0.0203 (7)	0.0171 (8)	0.0190 (7)	0.0001 (6)	0.0032 (6)	0.0003 (6)
C2	0.0263 (8)	0.0204 (8)	0.0213 (7)	0.0027 (7)	0.0077 (6)	−0.0029 (6)
C3	0.0218 (8)	0.0180 (8)	0.0323 (9)	0.0036 (6)	0.0085 (6)	−0.0017 (7)
C4	0.0192 (8)	0.0198 (8)	0.0284 (8)	0.0008 (6)	−0.0002 (6)	0.0014 (6)
C5	0.0205 (7)	0.0189 (8)	0.0213 (7)	−0.0007 (6)	−0.0004 (6)	−0.0010 (6)
C6	0.0154 (7)	0.0146 (7)	0.0192 (7)	−0.0014 (5)	0.0031 (5)	−0.0004 (6)
C7	0.0178 (7)	0.0158 (7)	0.0172 (7)	−0.0015 (6)	0.0021 (5)	−0.0011 (6)
C8	0.0156 (7)	0.0130 (7)	0.0210 (7)	−0.0012 (5)	0.0044 (5)	−0.0013 (6)
C9	0.0190 (7)	0.0159 (8)	0.0241 (8)	0.0005 (6)	0.0015 (6)	0.0007 (6)
C10	0.0198 (8)	0.0162 (8)	0.0322 (9)	0.0007 (6)	0.0006 (6)	0.0029 (7)
C11	0.0186 (7)	0.0136 (8)	0.0353 (9)	−0.0004 (6)	0.0089 (6)	−0.0003 (7)
C12	0.0274 (8)	0.0186 (8)	0.0249 (8)	−0.0044 (7)	0.0096 (6)	−0.0049 (6)
C13	0.0215 (8)	0.0184 (8)	0.0200 (7)	−0.0014 (6)	0.0058 (6)	−0.0026 (6)
C14	0.0194 (8)	0.0139 (8)	0.0426 (10)	−0.0005 (6)	0.0083 (7)	0.0010 (7)
C15	0.0304 (9)	0.0222 (9)	0.0286 (9)	−0.0042 (7)	0.0081 (7)	−0.0068 (7)
C16	0.0235 (8)	0.0243 (9)	0.0289 (8)	−0.0010 (7)	0.0002 (7)	−0.0009 (7)
C17	0.0195 (8)	0.0185 (8)	0.0310 (9)	0.0039 (6)	0.0065 (6)	−0.0006 (7)
C18	0.0278 (9)	0.0217 (9)	0.0268 (8)	−0.0008 (7)	0.0027 (6)	0.0013 (7)
C19	0.0215 (8)	0.0198 (9)	0.0376 (10)	−0.0002 (6)	0.0002 (7)	0.0062 (7)

### Geometric parameters (Å, °)

**Table:**

O1—C1	1.345 (2)	C8—C9	1.395 (2)
O1—H1O1	1.01 (3)	C8—C13	1.402 (2)
N1—C7	1.290 (2)	C9—C10	1.382 (2)
N1—C8	1.4146 (19)	C9—H9A	0.9300
N2A—N3A	1.264 (2)	C10—C11	1.383 (2)
N2A—C11	1.430 (2)	C10—H10A	0.9300
N3A—C14	1.437 (2)	C11—C12	1.406 (3)
N2B—N3B	1.292 (17)	C12—C13	1.390 (2)
N2B—C11	1.584 (12)	C12—H12A	0.9300
N3B—C14	1.655 (12)	C13—H13A	0.9300
C1—C2	1.400 (2)	C14—C19	1.377 (3)
C1—C6	1.409 (2)	C14—C15	1.404 (3)
C2—C3	1.384 (3)	C15—C16	1.398 (2)
C2—H2A	0.9300	C15—H15A	0.9300
C3—C4	1.391 (2)	C16—C17	1.390 (2)
C3—H3A	0.9300	C16—H16A	0.9300
C4—C5	1.383 (2)	C17—C18	1.388 (3)
C4—H4A	0.9300	C17—H17A	0.9300
C5—C6	1.400 (2)	C18—C19	1.379 (2)
C5—H5A	0.9300	C18—H18A	0.9300
C6—C7	1.449 (2)	C19—H19A	0.9300
C7—H7A	0.9300		
			
C1—O1—H1O1	106.4 (15)	C9—C10—H10A	119.9
C7—N1—C8	121.88 (13)	C10—C11—C12	119.47 (15)
N3A—N2A—C11	113.87 (16)	C10—C11—N2A	113.52 (16)
N2A—N3A—C14	112.55 (17)	C12—C11—N2A	127.01 (16)
N3B—N2B—C11	94.1 (9)	C10—C11—N2B	152.5 (4)
N2B—N3B—C14	93.0 (9)	C12—C11—N2B	88.0 (4)
O1—C1—C2	118.98 (14)	N2A—C11—N2B	39.1 (4)
O1—C1—C6	121.03 (14)	C13—C12—C11	120.14 (15)
C2—C1—C6	119.99 (15)	C13—C12—H12A	119.9
C3—C2—C1	119.58 (15)	C11—C12—H12A	119.9
C3—C2—H2A	120.2	C12—C13—C8	120.20 (16)
C1—C2—H2A	120.2	C12—C13—H13A	119.9
C2—C3—C4	121.16 (15)	C8—C13—H13A	119.9
C2—C3—H3A	119.4	C19—C14—C15	120.13 (16)
C4—C3—H3A	119.4	C19—C14—N3A	114.16 (16)
C5—C4—C3	119.28 (16)	C15—C14—N3A	125.71 (17)
C5—C4—H4A	120.4	C19—C14—N3B	155.6 (5)
C3—C4—H4A	120.4	C15—C14—N3B	84.2 (4)
C4—C5—C6	121.14 (15)	N3A—C14—N3B	41.5 (4)
C4—C5—H5A	119.4	C16—C15—C14	119.43 (17)
C6—C5—H5A	119.4	C16—C15—H15A	120.3
C5—C6—C1	118.81 (14)	C14—C15—H15A	120.3
C5—C6—C7	119.53 (14)	C17—C16—C15	119.70 (17)
C1—C6—C7	121.66 (14)	C17—C16—H16A	120.2
N1—C7—C6	121.13 (14)	C15—C16—H16A	120.2
N1—C7—H7A	119.4	C18—C17—C16	120.02 (16)
C6—C7—H7A	119.4	C18—C17—H17A	120.0
C9—C8—C13	118.75 (14)	C16—C17—H17A	120.0
C9—C8—N1	116.01 (14)	C19—C18—C17	120.46 (17)
C13—C8—N1	125.24 (14)	C19—C18—H18A	119.8
C10—C9—C8	121.15 (15)	C17—C18—H18A	119.8
C10—C9—H9A	119.4	C14—C19—C18	120.25 (17)
C8—C9—H9A	119.4	C14—C19—H19A	119.9
C11—C10—C9	120.29 (16)	C18—C19—H19A	119.9
C11—C10—H10A	119.9		
			
C11—N2A—N3A—C14	−179.52 (13)	N3A—N2A—C11—N2B	−0.3 (6)
C11—N2B—N3B—C14	179.1 (5)	N3B—N2B—C11—C10	3.7 (14)
O1—C1—C2—C3	−179.29 (16)	N3B—N2B—C11—C12	−178.6 (7)
C6—C1—C2—C3	0.0 (3)	N3B—N2B—C11—N2A	−0.4 (5)
C1—C2—C3—C4	1.3 (3)	C10—C11—C12—C13	−0.2 (3)
C2—C3—C4—C5	−1.4 (3)	N2A—C11—C12—C13	179.56 (16)
C3—C4—C5—C6	0.2 (3)	N2B—C11—C12—C13	−179.0 (4)
C4—C5—C6—C1	1.1 (2)	C11—C12—C13—C8	−0.1 (2)
C4—C5—C6—C7	−178.82 (15)	C9—C8—C13—C12	0.6 (2)
O1—C1—C6—C5	178.15 (15)	N1—C8—C13—C12	179.81 (14)
C2—C1—C6—C5	−1.2 (2)	N2A—N3A—C14—C19	−179.48 (15)
O1—C1—C6—C7	−2.0 (2)	N2A—N3A—C14—C15	0.2 (3)
C2—C1—C6—C7	178.71 (15)	N2A—N3A—C14—N3B	−0.7 (6)
C8—N1—C7—C6	−179.47 (13)	N2B—N3B—C14—C19	2.8 (15)
C5—C6—C7—N1	178.11 (15)	N2B—N3B—C14—C15	−179.1 (8)
C1—C6—C7—N1	−1.8 (2)	N2B—N3B—C14—N3A	0.1 (5)
C7—N1—C8—C9	−174.95 (15)	C19—C14—C15—C16	−0.8 (3)
C7—N1—C8—C13	5.8 (2)	N3A—C14—C15—C16	179.45 (16)
C13—C8—C9—C10	−0.9 (2)	N3B—C14—C15—C16	−179.9 (4)
N1—C8—C9—C10	179.83 (14)	C14—C15—C16—C17	−0.1 (3)
C8—C9—C10—C11	0.6 (3)	C15—C16—C17—C18	0.8 (3)
C9—C10—C11—C12	0.0 (3)	C16—C17—C18—C19	−0.6 (3)
C9—C10—C11—N2A	−179.87 (15)	C15—C14—C19—C18	1.0 (3)
C9—C10—C11—N2B	177.3 (9)	N3A—C14—C19—C18	−179.21 (15)
N3A—N2A—C11—C10	−178.25 (15)	N3B—C14—C19—C18	178.8 (10)
N3A—N2A—C11—C12	1.9 (3)	C17—C18—C19—C14	−0.3 (3)

### Hydrogen-bond geometry (Å, °)

**Table:**

*D*—H···*A*	*D*—H	H···*A*	*D*···*A*	*D*—H···*A*
O1—H1O1···N1	1.01 (3)	1.66 (2)	2.5853 (18)	150 (2)
C5—H5A···O1^i^	0.93	2.59	3.386 (2)	144

Symmetry codes: (i) *x*, −*y*−1/2, *z*+1/2.
